# Oxidative Stress in Chronic Rhinosinusitis with Nasal Polyps: Pathophysiological Mechanisms, Biomarkers, and Therapeutic Implications

**DOI:** 10.3390/medicina62101910

**Published:** 2026-10-03

**Authors:** Cosimo Galletti, Mariapia Giacoppo, Edoardo Venticinque, Jerome Rene Lechien, Federica Maria Parisi, Mario Lentini, Christian Calvo-Henriquez, Juan Maza-Solano, Salvatore Maira, Antonino Maniaci

**Affiliations:** 1 Faculty of Medicine and Surgery, University of Enna ‘Kore’, 94100 Enna, Italy; cosimo.galletti01@unikore.it (C.G.); edoardo.venticinque@unikorestudent.it (E.V.);; 2 Department of Anatomy and Experimental Oncology, University of Mons (UMONS), 7000 Mons, Belgium; 3 Department of Otorhinolaryngology, Complejo Hospitalario Universitario de Santiago de Compostela, 15706 Santiago de Compostela, Spain; 4 Rhinology and Skull Base Unit, Department of Otorhinolaryngology, University Hospital Virgen del Rocio, 41001 Seville, Spain; 5 Department of Surgery, University of Seville, 41001 Seville, Spain

**Keywords:** chronic rhinosinusitis with nasal polyps, CRSwNP, oxidative stress, reactive oxygen species, antioxidants, epithelial barrier dysfunction, type 2 inflammation, endotype, asthma, allergic rhinitis, united airways disease

## Abstract

*Background and Objectives*: Chronic rhinosinusitis with nasal polyps (CRSwNP) is a heterogeneous inflammatory disease of the sinonasal mucosa characterized by persistent symptoms, high recurrence rates, and a significant impact on quality of life. Increasing evidence suggests that oxidative stress plays a role in its pathophysiology, although its precise contribution remains incompletely defined. *Materials and Methods*: A narrative review was conducted following SANRA recommendations. A structured literature search was performed using PubMed, Scopus, and Google Scholar to identify studies investigating oxidative stress, antioxidant systems, and molecular pathways in CRSwNP. Relevant articles published between January 2000 and March 2026 were included and critically appraised, distinguishing clinical from ex vivo, in vitro, and animal evidence. *Results*: Available evidence, derived mainly from small cross-sectional case–control studies, consistently associates CRSwNP with increased oxidative stress, characterized by elevated levels of reactive oxygen and nitrogen species, lipid peroxidation products, and DNA damage. Impaired antioxidant defenses accompany this imbalance, although findings for individual enzymes such as superoxide dismutase, catalase, and glutathione peroxidase are inconsistent across sample types and assays. At the molecular level, oxidative stress activates pro-inflammatory signalling pathways, including NF-κB and the NLRP3 inflammasome, and is linked to epithelial barrier dysfunction and type 2 immune responses. Oxidative signatures appear to differ between eosinophilic and non-eosinophilic endotypes. Furthermore, oxidative stress represents a plausible mechanistic link between CRSwNP, asthma, and allergic rhinitis, supporting the concept of united airways disease. Antioxidant strategies are supported almost exclusively by preclinical data. *Conclusions*: Oxidative stress is a consistent feature of CRSwNP and, on the basis of experimental data, a likely amplifier of chronic inflammation; however, a causal role in humans has not been established. Standardized, endotype-specific oxidative biomarkers, longitudinal studies, and randomized trials are needed before redox-targeted strategies can be integrated into clinical practice.

## 1. Introduction

Chronic rhinosinusitis with nasal polyps (CRSwNP) is defined as a chronic inflammatory condition of the sinonasal mucosa characterized by signs and symptoms that persist for more than 12 weeks and the development of bilateral nasal polypoid lesions that can be visualized through nasal endoscopy [1,2]. CRSwNP causes significant mucosal swelling that leads to nasal obstruction and loss of smell, with significant morbidity and decreased quality of life. Medical and surgical treatments have improved patient outcomes, but recurrence remains high and there is still a significant long-term burden of illness [1].

CRSwNP has been estimated to occur in approximately 0.5–4% of the population and accounts for up to 25–30% of all CRS cases [3]. This condition is usually seen in adults and is commonly associated with asthma and allergic rhinitis. In Western countries, CRSwNP is most commonly associated with type 2 inflammation (eosinophilic endotype), which is linked with increased severity and recurrence [4], whereas East Asian cohorts include a larger proportion of non-eosinophilic, neutrophil-rich, or mixed inflammatory patterns, although the prevalence of eosinophilic disease is increasing in this region [5,6].

Histologically, CRSwNP is complex and heterogeneous, driven by epithelial barrier defects, dysregulated immune responses, and tissue remodeling [7,8]. The sinonasal epithelium serves as the first line of defense against inhaled foreign materials, acting as both a physical barrier and an immune regulator. Disruption of epithelial barriers allows for greater exposure of the mucosa to environmental agents such as allergens, pollutants, and microbes, leading to polarization of a type 2 immune response with cytokines such as IL-5 and IL-13 [7,8]. Chronic inflammation also drives extracellular matrix remodeling and stromal edema that culminate in polyp formation. A role for oxidative stress in CRSwNP has recently been proposed. Oxidative stress is described as an imbalance between the production of reactive oxygen species (ROS) and reactive nitrogen species (RNS) and the available antioxidant defenses [9]. While ROS play critical roles in normal cell signaling and pathogen defense, uncontrolled oxidative species lead to lipid peroxidation, protein oxidation, DNA damage, and perpetuation of inflammatory signaling cascades [9].

The sinonasal mucosa is constantly exposed to environmental oxidative stimuli from air pollution, cigarette smoke, and microbial virulence factors. In CRSwNP, which is characterized by epithelial dysfunction and chronic inflammation, a vicious cycle may therefore be established, in which oxidative species cause continued tissue damage and, in turn, sustain inflammation [7,9].

Clinical studies in patients with nasal polyps have reported increased oxidative stress markers, such as plasma nitric oxide, total oxidant status, and oxidative stress index [10,11], as well as increased oxidative DNA damage in circulating lymphocytes [10]. Reduced activities of antioxidant enzymes, such as superoxide dismutase, catalase, and glutathione peroxidase, have also been described, although these reductions have not consistently reached statistical significance [10,12]. Transcriptomic analyses of nasal polyp tissue have also confirmed dysregulation of oxidative stress and antioxidant pathways involved in redox homeostasis [13,14].

Although clinical evidence supports the existence of oxidative stress in CRSwNP, much remains to be understood about the role that it plays. It is currently unclear whether oxidative stress represents a disease-driving mechanism or merely an epiphenomenon of the inflammatory process. Studies evaluating oxidative stress in CRSwNP are heterogeneous in the biomarkers analyzed, experimental methods, and patient subgrouping, and most are cross-sectional. Additionally, there are limited data assessing the longitudinal impact and clinical utility of oxidative stress biomarkers [9].

Thus, a comprehensive review of the literature is needed to better summarize the current evidence surrounding oxidative stress in CRSwNP. The objective of this narrative review is to analyze the evidence on oxidative stress and its role in nasal polyps, discussing underlying mechanisms, biomarkers, and therapeutic implications, and to critically appraise the strength of the available evidence by distinguishing clinical from preclinical data and by examining differences across CRSwNP endotypes and populations.

## 2. Materials and Methods

### 2.1. Study Design

We set out to provide an exhaustive yet critical examination of the literature available on the involvement of oxidative stress in CRSwNP. The methodology used to prepare this narrative review was designed based on recommendations provided by the Scale for the Assessment of Narrative Review Articles (SANRA), with particular focus on the disclosure, literature selection, and article critique domains. The review aimed to integrate findings from basic science, translational research, and clinical studies in order to explore the biological relevance and potential clinical implications of oxidative stress in CRSwNP.

### 2.2. Literature Search Strategy

A structured literature search was performed using the electronic databases PubMed, Scopus, and Google Scholar. The search covered publications from January 2000 to March 2026.

The following keywords and Medical Subject Headings (MeSH) terms were used in various combinations: “chronic rhinosinusitis with nasal polyps”, “CRSwNP”, “oxidative stress”, “reactive oxygen species”, “antioxidant”, “redox imbalance”, “nasal polyps”, “lipid peroxidation”, “NRF2”, “eosinophilic”, and “endotype”. Boolean operators (AND, OR) were applied to refine the search strategy.

In addition, the reference lists of relevant articles were manually screened to identify further eligible studies, ensuring a comprehensive inclusion of the available literature [15,16].

### 2.3. Eligibility Criteria

Studies were considered eligible if they met the following criteria:

(i) Original research articles, reviews, or meta-analyses focusing on oxidative stress in CRSwNP or closely related CRS phenotypes;
(ii) studies investigating oxidative biomarkers, antioxidant systems, or redox-related molecular pathways;
(iii) articles published in peer-reviewed journals in English.

Exclusion criteria included case reports, editorials without original data, conference abstracts lacking full text, and studies not specifically addressing oxidative stress mechanisms in sinonasal disease [15,17].

### 2.4. Study Selection and Data Extraction

Study selection included initial title and abstract screening, followed by full-text screening of potentially eligible studies. We took into consideration methodological quality, study design, and relevance to the review question. Extracted data included study characteristics (authors, year of publication, and study design), patient population and phenotype or endotype (when reported), sample type (serum or plasma, erythrocytes, whole tissue, or isolated epithelial cells), analytical method, oxidative stress markers evaluated, and main findings concerning redox imbalance in CRSwNP. Whenever available, we also extracted information about antioxidant enzyme activities and molecular pathways to provide a more comprehensive interpretation of the data [16,17].

### 2.5. Data Synthesis and Critical Appraisal

Due to substantial heterogeneity in study design, biomarkers, sample types, and outcome measures, we did not pursue a quantitative meta-analysis. Instead, we conducted a qualitative synthesis based on consistency of results, biological plausibility, and clinical importance. Risk of bias and effect modifiers were considered qualitatively by assessing sample size, type of control group, analytical method, and differences in patient phenotyping. Evidence was also classified by study type, distinguishing between human observational studies, ex vivo human tissue studies, in vitro studies, animal models, and interventional clinical trials; the main clinical studies are summarized in Table 1 and the therapeutic evidence, stratified by level, in Table 2. Special consideration was given to whether oxidative stress is causal or consequential in the pathogenesis of CRSwNP [15,16,17].

## 3. Results

### 3.1. Oxidative Biomarkers in CRSwNP

Several studies have sought to identify biomarkers that reliably reflect systemic or local redox imbalance in CRSwNP. Overall, the available evidence consistently indicates that patients with CRSwNP present altered systemic and local oxidative biomarkers, although it derives mainly from small, single-center, cross-sectional case–control studies (Table 1). In nasal polyp tissue, immunohistochemistry has shown increased staining for 4-hydroxynonenal (4-HNE), a lipid peroxidation end-product, compared with control mucosa [14], and increased lipid hydroperoxide levels have been measured in both serum and polyp tissue [12]. Because they reflect oxidative damage to cell membranes, reactive aldehydes are among the most widely studied markers of oxidative injury [9]. Furthermore, increased protein nitration, documented by 3-nitrotyrosine staining, has been observed in nasal polyps, indicating oxidative injury mediated by reactive nitrogen species [14].

Nitric oxide (NO) also plays an important role in the pathophysiology of CRSwNP. While involved in normal physiological processes such as host defense and mucociliary clearance, its sustained overproduction appears to contribute to disease activity, and increased plasma NO concentrations have been reported in patients with nasal polyps [10]. A proteomic study identified an upregulation of NO-mediated signaling in polyp tissue and found that soluble guanylate cyclase subunits (GUCY1A1 and GUCY1A2) predicted postoperative recurrence in two independent cohorts [18]. These findings have raised interest in NO-pathway markers as prognostic biomarkers, although external validation is still lacking.

Alterations in systemic oxidative biomarkers have also been reported. Patients with nasal polyps presented increased total oxidant status (TOS) and oxidative stress index (OSI) and decreased total antioxidant status (TAS) in serum and, in one study, in polyp tissue as well [11,19]. It is worth noting that these studies used either healthy volunteers or patients undergoing septal or turbinate surgery as controls, and did not adjust for smoking, atopy, or corticosteroid use, which limits comparability.

Further studies have focused on transcriptomic and molecular markers in CRSwNP tissue. Using a targeted oxidative stress gene array in 25 patients and 25 controls, Tsai et al. found inducible nitric oxide synthase (iNOS) and heme oxygenase-1 (HMOX1) to be upregulated, whereas superoxide dismutase 3 (SOD3) and the heme peroxidases lactoperoxidase (LPO) and myeloperoxidase (MPO) were downregulated in polyp tissue [14]. Because MPO is itself an oxidant-generating enzyme, these changes indicate a broad remodeling of redox-related gene expression rather than a simple loss of antioxidant capacity. Bioinformatic analyses combining public datasets with machine learning have proposed additional oxidative stress-related hub genes with diagnostic potential [20], and Mihalj et al. showed that transcripts of the antioxidant peroxiredoxin-2 scaled with radiological disease severity and were only variably normalized after surgery and corticosteroid therapy [13].

Initial evidence also suggests that oxidative stress-related biomarkers could contribute to disease stratification, with candidate markers of severity or recurrence including redox-related gene signatures and NO-pathway proteins [13,18]. Together, these data support the hypothesis that redox imbalance participates in CRSwNP pathophysiology; however, no oxidative biomarker has yet been validated for clinical use. Heterogeneity in sample type (serum, plasma, erythrocytes, whole tissue, or isolated epithelial cells), analytical method (spectrophotometric composite indices, ELISA, immunohistochemistry, PCR arrays, or sequencing), control groups, and patient phenotyping, together with the absence of agreed thresholds, has so far prevented clinical implementation (Table 1).

**Table 1:** Main human studies evaluating oxidative stress in CRSwNP: design, samples, findings, and limitations.

Study	Design and Population	Sample and Method	Main Oxidative Stress Findings	Main Limitations
Hao et al., 2013 [11]	Prospective case–control; 32 NP vs. 29 healthy controls (China)	Serum; PON, ARE, TAS, TOS, OSI (spectrophotometry)	↑ TOS and OSI; ↓ TAS, PON, and ARE	Serum only; no endotyping; single center
Bozkus et al., 2013 [19]	Cross-sectional; 38 NP vs. 35 surgical controls (septal deviation/turbinate hypertrophy) (Turkey)	Serum and tissue; TOS, TAS, OSI	↑ TOS and OSI, ↓ TAS in serum and tissue; tissue > serum	Non-healthy controls; composite indices; no endotyping
San et al., 2013 [12]	Cross-sectional; 30 NP vs. 30 surgical controls (Turkey)	Serum and tissue; LOOH, CAT, prolidase	↑ LOOH and ↓ CAT in serum and tissue; correlation with prolidase	Non-healthy controls; no endotyping
Mrowicka et al., 2015 [10]	Case–control; 20 NP vs. 20 healthy controls (Poland)	Erythrocyte SOD, CAT, GPx; plasma NO; lymphocyte comet assay	↑ NO and oxidative DNA damage; non-significant ↓ SOD, CAT, GPx	Very small sample; systemic markers only
Yu et al., 2015 [21]	Case–control; 40 CRSwNP (17 eosinophilic, 23 non-eosinophilic) vs. 20 controls (China)	Tissue HO-1 (qRT-PCR, IHC, Western blot); explant stimulation	↑ HO-1 in polyps; higher in non-eosinophilic than in eosinophilic CRSwNP	Single marker; small subgroups
Akyigit et al., 2017 [22]	Genetic association; 130 CRSwNP vs. 188 controls (Turkey)	Blood DNA; SOD2, CAT, and iNOS polymorphisms	CAT and iNOS genotypes associated with eosinophilic CRSwNP	Candidate-gene design; no replication
Yoon et al., 2020 [23]	Case–control plus in vitro; 30 CRSwNP vs. 15 controls (Korea)	Tissue and nasal epithelial cells; mitochondrial ROS, OXPHOS complexes, electron microscopy	↑ mitochondrial ROS and altered mitochondrial dynamics in polyp epithelial cells	Small sample; mechanism shown in vitro
Mihalj et al., 2022 [13]	Observational, with sampling before and after treatment (Croatia)	Tissue mRNA (SOD, PRDX2, and innate defense genes)	PRDX2 transcripts scaled with radiological severity; variable normalization after surgery and corticosteroids	Transcript level only; no endotyping
Tsai et al., 2022 [14]	Case–control; 25 CRSwNP vs. 25 controls (Taiwan)	Tissue IHC (4-HNE, 3-nitrotyrosine); 84-gene PCR array; Western blot	↑ 4-HNE and 3-nitrotyrosine; ↑ iNOS and HO-1; ↓ SOD3, LPO, and MPO	Targeted array; no endotyping
Zhou et al., 2024 [24]	Tissue study plus in vitro and explant experiments (China)	IHC, immunofluorescence, qPCR; epithelial cells and macrophages	↑ NOS2, NOX1, HO-1, and SOD2; oxidases correlated with IL-5 and IL-6	Correlational tissue data; preclinical interventions
Jiang et al., 2024 [25]	Single-cell RNA-seq reanalysis plus tissue and in vitro study (China)	Epithelial ROS, NOX2, NLRP3 inflammasome	↑ NOX2–ROS–NLRP3 axis and alarmins in CRSwNP epithelium	Public dataset; mechanism shown in vitro
Jiang et al., 2025 [18]	Discovery and two validation cohorts (China)	Tissue protein array, Western blot, IHC	NO–sGC pathway upregulated; GUCY1A1/GUCY1A2 predicted recurrence	No external validation
Park et al., 2025 [26]	Bulk and single-cell RNA-seq plus air–liquid interface cultures (Korea)	Epithelial lipid peroxidation genes (SLC27A2/FATP2)	IL-4/IL-13-driven lipid peroxidation; SLC27A2 correlated with severity in eosinophilic CRSwNP; reduced by dupilumab	Transcriptomic; functional data in vitro
Zhang et al., 2025 [27]	Tissue study plus in vitro experiments; eosinophilic vs. non-eosinophilic CRSwNP (China)	NLRP3, IL-1β, cleaved GSDMD, caspase-1	Pyroptosis in both endotypes, stronger in non-eosinophilic CRSwNP; IL-5 vs. IL-17A as triggers	Small cohorts
Gu et al., 2026 [28]	RNA-seq; 9 eosinophilic and 5 non-eosinophilic CRSwNP vs. 8 controls (China)	Tissue transcriptome with external and tissue validation	↑ NCF2, NCF4, RAC2, HIF1A, and SELP in eosinophilic CRSwNP	Very small discovery cohort

↑, increased; ↓, decreased; 4-HNE, 4-hydroxynonenal; ARE, arylesterase; CAT, catalase; GPx, glutathione peroxidase; GSDMD, gasdermin D; HO-1, heme oxygenase-1; IHC, immunohistochemistry; iNOS, inducible nitric oxide synthase; LOOH, lipid hydroperoxides; LPO, lactoperoxidase; MPO, myeloperoxidase; NO, nitric oxide; NP, nasal polyps; OSI, oxidative stress index; OXPHOS, oxidative phosphorylation; PON, paraoxonase; PRDX2, peroxiredoxin-2; sGC, soluble guanylate cyclase; SOD, superoxide dismutase; TAS, total antioxidant status; TOS, total oxidant status.

### 3.2. Antioxidant Systems in CRSwNP

Antioxidant enzymes represent the first line of enzymatic defense against ROS and operate both independently and synergistically. Superoxide dismutase (SOD) converts superoxide radicals into hydrogen peroxide, which is then removed by catalase (CAT), yielding water and oxygen, and by glutathione peroxidase (GPx), which reduces it to water at the expense of reduced glutathione [9,29]. Available data suggest that this antioxidant capacity is impaired in CRSwNP, although findings vary across compartments and assays.

Reduced catalase activity has been reported in both serum and polyp tissue [12], and total antioxidant status is lower in the serum and tissue of patients with nasal polyps [11,19]. In contrast, erythrocyte SOD, CAT, and GPx activities showed only non-significant reductions in another cohort [10], and tissue expression of some antioxidant enzymes, such as SOD2 and HO-1, was actually increased, probably as an adaptive response to oxidative load [14,24]. Downregulation of glutathione metabolism-related proteins has been described in the nasal secretions of children with CRSwNP [30], whereas data on glutathione in adults remain limited.

NRF2 is the master transcriptional regulator of the antioxidant response, driving antioxidant response element-dependent expression of antioxidant enzymes, such as SOD and CAT, as well as phase II detoxification enzymes. Evidence for its role in sinonasal inflammation is mainly experimental: deletion of NRF2, either whole-body or restricted to the sinonasal epithelium, enhanced eosinophilic inflammation, epithelial alarmin release (IL-33 and TSLP), goblet cell hyperplasia, and barrier dysfunction in murine models of rhinosinusitis [31,32], whereas pharmacological NRF2 activation prevented allergen- and cigarette smoke-induced barrier disruption in human sinonasal epithelial cultures [33,34]. Insufficient NRF2 responsiveness may therefore contribute to uncontrolled oxidative stress and inflammatory cytokine release, although direct evidence of reduced NRF2 activity in patients with CRSwNP remains limited [9].

In polyp tissue, the expression of oxidases (NOS2 and NOX1) correlates positively with inflammatory cytokines such as IL-5 and IL-6, whereas the levels of antioxidant enzymes correlate negatively with IL-13 and IFN-γ; nasal epithelial cells and macrophages have been identified as the main cellular sources of oxidative stress [24].

Experimental antioxidant interventions have been shown to reduce sinonasal inflammation and oxidative stress in cellular, explant, and animal models [24,33,35]. These data provide a rationale for antioxidants as potential adjuvant treatments; however, to date no randomized controlled trial has evaluated antioxidant therapy in patients with CRSwNP (Section 3.4 and Table 2).

### 3.3. Molecular Pathways Linking Oxidative Stress and Inflammation

The interaction between oxidative stress and inflammatory signaling pathways represents a pivotal mechanism in the pathogenesis of chronic rhinosinusitis with nasal polyps (CRSwNP). Experimental evidence suggests that reactive oxygen species (ROS) are not merely by-products of inflammation but active modulators of intracellular signaling cascades that amplify and sustain the inflammatory response.

One of the central molecular pathways involved is the activation of nuclear factor kappa B (NF-κB), a key transcription factor regulating the expression of pro-inflammatory cytokines, adhesion molecules, and chemokines. Oxidative stress has been shown, mainly in vitro, to induce NF-κB activation within nasal epithelial cells, leading to enhanced transcription of inflammatory mediators and perpetuation of mucosal inflammation [9]. This process may contribute to a chronic inflammatory loop in which ROS production and cytokine signaling reinforce each other.

In parallel, oxidative stress interacts with the type 2 inflammatory microenvironment characteristic of CRSwNP. IL-4 and IL-13 induce epithelial 15-lipoxygenase-1 (ALOX15) and lipid peroxidation programs [26,36], and IL-5 has been identified as a trigger of NLRP3-dependent pyroptosis in eosinophilic polyps [27]. Conversely, the expression of oxidases such as NOS2 and NOX1 correlates with IL-5 levels in polyp tissue [24]. These data suggest a bidirectional relationship, in which oxidative stress enhances type 2 inflammation while type 2 cytokines further increase oxidant production, although most of this evidence is correlative or derived from in vitro models.

At the epithelial level, oxidative stress contributes to barrier dysfunction, which represents a hallmark of CRSwNP. ROS-mediated damage to tight junction proteins leads to increased epithelial permeability, facilitating the penetration of allergens and microbial agents [7,33,34]. This disruption triggers the release of epithelial-derived cytokines, including thymic stromal lymphopoietin (TSLP), IL-25, and IL-33, which are key initiators of type 2 immune responses [8,31]. Consequently, epithelial damage is not merely a structural alteration but an active driver of immune dysregulation.

Recent molecular studies have further identified the involvement of NADPH oxidase systems, particularly NOX2, in ROS generation within nasal epithelial cells. Increased NOX2 expression in CRSwNP epithelium has been associated with ROS accumulation, activation of the NLRP3 inflammasome, and epithelial alarmin expression, and NOX2 silencing reversed these effects in vitro [25]. This pathway promotes the release of pro-inflammatory cytokines such as IL-1β and contributes to epithelial inflammation and remodeling. Mitochondria represent an additional source of ROS: mitochondrial ROS and altered mitochondrial dynamics were increased in polyp epithelial cells and were induced by Staphylococcus aureus enterotoxin B in nasal epithelial cells [23]. The activation of inflammasome pathways provides an additional link between oxidative stress and both innate and adaptive immune responses.

Moreover, oxidative stress-related inflammasome activation has been implicated in the dysregulation of epithelial differentiation and repair. Pyroptosis impairs the differentiation of basal cells, favoring goblet cell differentiation, mucus hypersecretion, and reduced mucociliary clearance, and its inhibition restored the balance of basal cell differentiation in vitro [27]. These changes further exacerbate the inflammatory milieu and contribute to the persistence of sinonasal disease.

**Figure 1:**
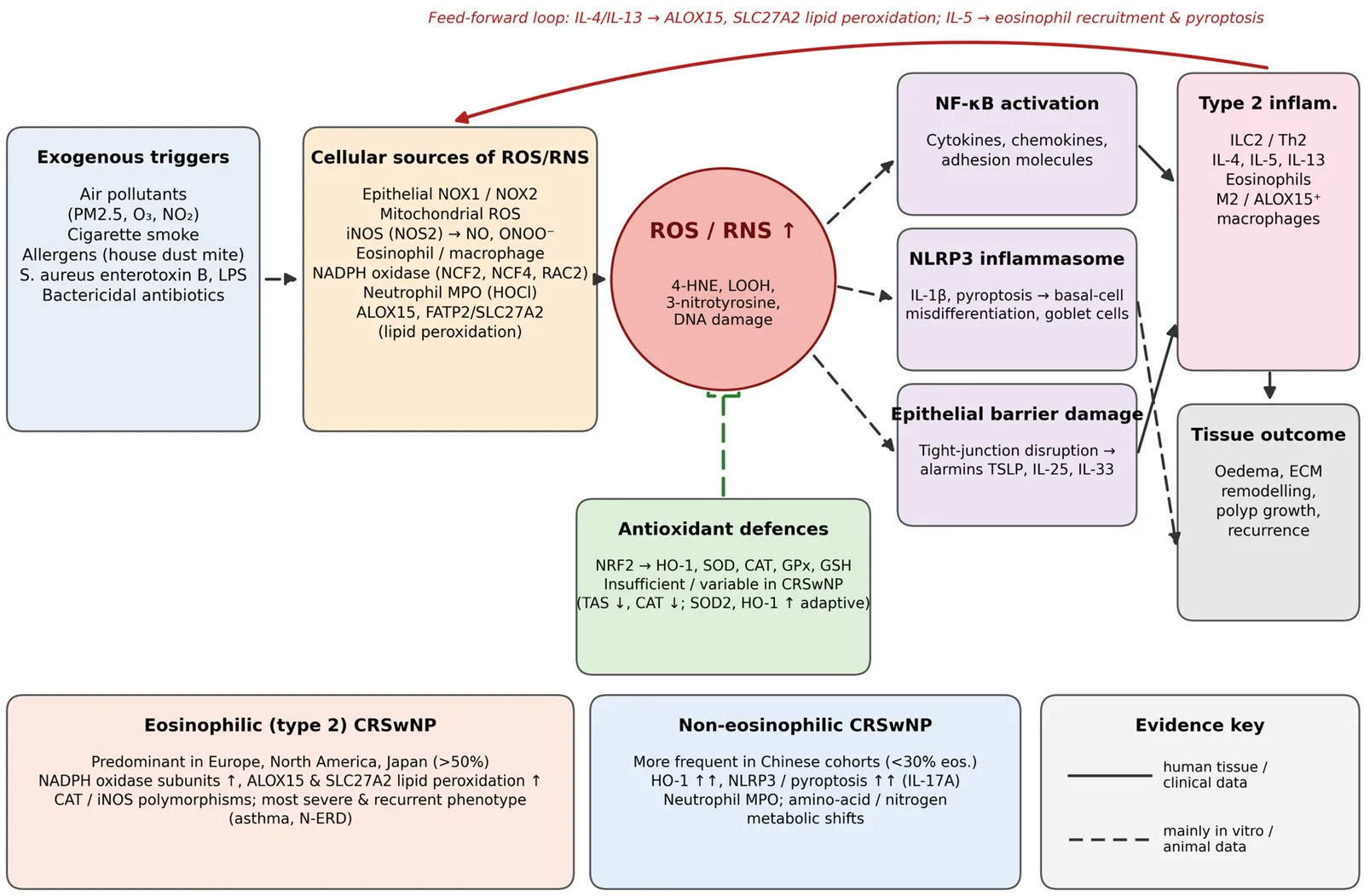
Schematic representation of the proposed pathways linking oxidative stress and chronic inflammation in CRSwNP. Exogenous triggers and endogenous cellular sources (epithelial NOX1/NOX2, mitochondria, iNOS, eosinophil/macrophage NADPH oxidase, neutrophil myeloperoxidase, and ALOX15/FATP2-dependent lipid peroxidation) increase ROS/RNS, which are insufficiently counterbalanced by NRF2-dependent antioxidant defenses. ROS/RNS activate NF-κB and the NLRP3 inflammasome and damage epithelial tight junctions, promoting alarmin release and type 2 inflammation, which in turn feeds back on oxidant production (feed-forward loop). The lower panels summarize the main differences between eosinophilic and non-eosinophilic endotypes. Solid arrows indicate links supported by human tissue or clinical data; dashed arrows indicate links supported mainly by in vitro or animal data. 4-HNE, 4-hydroxynonenal; ALOX15, arachidonate 15-lipoxygenase; CAT, catalase; GPx, glutathione peroxidase; GSH, reduced glutathione; HO-1, heme oxygenase-1; iNOS, inducible nitric oxide synthase; LOOH, lipid hydroperoxides; MPO, myeloperoxidase; N-ERD, NSAID-exacerbated respiratory disease; NOX, NADPH oxidase; RNS, reactive nitrogen species; ROS, reactive oxygen species; SOD, superoxide dismutase; TAS, total antioxidant status.

Taken together, these findings support a working model, derived predominantly from experimental data, in which oxidative stress acts as a hub connecting epithelial dysfunction, innate immune activation, and type 2 inflammation (Figure 1). Whether redox imbalance initiates the disease or mainly sustains it once inflammation is established cannot be determined from the current human data.

### 3.4. Clinical Implications and Therapeutic Perspectives

The growing body of evidence linking oxidative stress to the pathophysiology of chronic rhinosinusitis with nasal polyps (CRSwNP) has potential clinical implications, particularly in terms of disease stratification and therapeutic targeting. Although causality has not been established in humans, experimental data suggest that redox imbalance may contribute to disease persistence, remodeling, and treatment resistance, thereby opening new perspectives for personalized management.

From a diagnostic standpoint, oxidative stress-related biomarkers may provide additional tools for patient stratification beyond traditional phenotypic classification. Emerging data suggest that increased expression of oxidative enzymes such as NOX and NOS, along with reduced antioxidant capacity, correlates with inflammatory burden, and that redox-related transcripts and NO-pathway proteins may track disease severity or recurrence [13,18,24]. Oxidative stress profiling could therefore help identify patients with more aggressive or refractory disease, although this remains hypothesis-generating. In this context, redox-related gene signatures may also contribute to endotype-driven approaches, complementing current inflammatory classifications (Section 3.6).

Therapeutically, current standard treatments, including intranasal corticosteroids, systemic steroids, and endoscopic sinus surgery, primarily target inflammation but do not directly address the underlying oxidative imbalance. In line with this, surgery followed by corticosteroid therapy only variably restored redox-related gene expression in the nasal mucosa [13], and bactericidal antibiotics, which are widely used in CRS, increased ROS production in sinonasal epithelial cells in vitro [37]. Persistent redox dysregulation might therefore contribute to the high recurrence rates observed in CRSwNP, although this hypothesis has not been tested directly.

Experimental studies have explored the potential role of antioxidant-based interventions. Compounds capable of modulating oxidative pathways, such as NRF2 activators, have reduced inflammatory responses and preserved epithelial barrier integrity in preclinical models [33,34]. For example, crocin enhanced antioxidant defenses via the KEAP1/NRF2/HO-1 pathway while suppressing pro-inflammatory signaling in nasal epithelial cells, macrophages, and polyp explants [24]. These results highlight the dual anti-inflammatory and cytoprotective potential of redox-targeted strategies, but they have not yet been tested in patients.

In addition to classical antioxidants, biologic agents targeting type 2 inflammation [4,38] may indirectly modulate oxidative stress. In transcriptomic analyses, IL-4 receptor α blockade with dupilumab reduced the expression of lipid peroxidation-associated genes, including SLC27A2, in nasal epithelial cells [26]. This suggests that modulation of inflammatory cascades may have secondary effects on redox balance; however, whether anti-IL-5/IL-5R or anti-IgE agents exert similar effects has not been investigated, and redox markers have not been included as endpoints in clinical trials of biologics.

Furthermore, early experimental approaches have investigated adjunctive compounds with combined antioxidant and anti-inflammatory properties. In an ex vivo study with within-patient randomization of polyp explants from 30 patients, boric acid reduced malondialdehyde and TOS, increased TAS and catalase activity, and attenuated type 2 cytokine expression, both alone and combined with triamcinolone [35]. These findings support further evaluation of redox-modulating agents as part of combination or steroid-sparing strategies, but they are limited to 72 h tissue cultures.

Table 2 summarizes the therapeutic evidence stratified by level. Importantly, human interventional data are almost absent: our search did not identify any randomized controlled trial of antioxidant therapy with clinical endpoints in CRSwNP. The only randomized data involving a thiol antioxidant come from recurrent acute rhinosinusitis, in which N-acetylcysteine was used as a mucoactive agent in combination with topical corticosteroids [39]; these results cannot be extrapolated to CRSwNP. Accordingly, antioxidant supplementation cannot currently be recommended for the treatment or prevention of CRSwNP outside of clinical trials.

**Table 2:** Therapeutic and interventional evidence on redox-targeted strategies in CRSwNP, stratified by level of evidence.

Level of Evidence	Intervention or Target	Model	Main Findings	Reference
In vitro	Sulforaphane (NRF2 activator)	Human sinonasal epithelial cells at air–liquid interface exposed to house dust mite or cigarette smoke extract	Prevented tight-junction disruption and preserved transepithelial resistance	[33,34]
In vitro	NOX2 silencing; NLRP3 inhibition (MCC950)	Human nasal epithelial cells	↓ ROS, NLRP3 activation, and alarmin expression	[25]
In vitro	MitoTEMPO (mitochondrial ROS scavenger)	Nasal epithelial cells stimulated with S. aureus enterotoxin B	↓ mitochondrial ROS	[23]
In vitro	FATP2 inhibition	Nasal epithelial cells at air–liquid interface	↓ IL13RA1 and lipid peroxidation-related genes	[26]
In vitro/ex vivo	Crocin (KEAP1/NRF2/HO-1 pathway)	Nasal epithelial cells, macrophages, polyp explants	↓ NOS2 and NOX1; ↓ inflammation; ↑ antioxidant capacity	[24]
Ex vivo	Boric acid ± triamcinolone	Polyp explants from 30 patients (72 h culture)	↓ MDA, TOS, and type 2 cytokines; ↑ TAS and CAT	[35]
Animal	Nrf2 deletion (whole-body or epithelium-specific)	Ovalbumin- or papain-induced murine rhinosinusitis	Aggravated eosinophilia, alarmin release, and barrier dysfunction (supports a protective role of NRF2)	[31,32]
Animal	ALOX15 inhibition	Murine eosinophilic rhinosinusitis	↓ type 2 immune overactivation	[40]
Human genetic	ALOX15 loss-of-function variant (p.Thr560Met)	Genome-wide association study (4366 NP cases)	Strong protection against NP (OR 0.32)	[41]
Human, indirect	Dupilumab (IL-4Rα blockade)	Transcriptomic analysis of nasal epithelium	↓ lipid peroxidation-associated genes, including SLC27A2	[26]
Human RCT	No antioxidant RCT in CRSwNP; N-acetylcysteine used as mucoactive agent in recurrent acute rhinosinusitis	Single-blind randomized study	Not transferable to CRSwNP	[39]

↑, increased; ↓, decreased; CAT, catalase; MDA, malondialdehyde; NP, nasal polyps; OR, odds ratio; RCT, randomized controlled trial; ROS, reactive oxygen species; TAS, total antioxidant status; TOS, total oxidant status.

Despite these promising developments, several challenges remain. Clinical translation is limited by the lack of standardized oxidative biomarkers, heterogeneity in study designs, insufficient longitudinal data assessing treatment response, and the near absence of human interventional studies. Moreover, it is still unclear which patient subgroups would benefit most from antioxidant-based interventions, emphasizing the need for more precise endotyping strategies.

Overall, the integration of oxidative stress into the clinical framework of CRSwNP represents a promising but still evolving field. Future research should aim to validate redox biomarkers in large cohorts and to evaluate targeted therapies in well-designed clinical trials, with the ultimate goal of improving disease control and reducing recurrence rates.

### 3.5. From Allergy and Asthma to Nasal Polyps: The Role of Oxidative Stress

The close relationship between allergic diseases, asthma, and chronic rhinosinusitis with nasal polyps (CRSwNP) is increasingly recognized within the framework of the “united airways disease” concept, in which upper and lower airway inflammation share common pathogenic mechanisms. In this continuum, oxidative stress emerges as a plausible link connecting allergic sensitization, bronchial inflammation, and sinonasal disease progression.

Allergic rhinitis and asthma are both characterized by type 2 immune responses and are strongly associated with increased production of reactive oxygen species (ROS). Environmental allergens, including house dust mites and airborne pollutants, can directly induce ROS generation in airway epithelial cells while simultaneously impairing antioxidant defenses, thereby amplifying inflammatory signaling [42,43]. In asthma, oxidative stress has been shown to promote airway hyperresponsiveness, mucus hypersecretion, and structural remodeling, all of which contribute to chronic disease persistence [43,44].

This redox imbalance is not confined to the lower airways. The sinonasal mucosa is similarly exposed to oxidative stimuli and exhibits comparable inflammatory responses. In patients with CRSwNP, increased oxidative stress has been demonstrated in nasal epithelial cells and polyp tissue, where excessive ROS production is associated with mitochondrial dysfunction, epithelial damage, and altered cellular metabolism [14,23]. These alterations mirror those observed in asthma, further supporting the concept of a shared pathophysiological axis.

Clinically, the coexistence of asthma and CRSwNP is associated with a more severe disease phenotype, characterized by increased symptom burden, higher recurrence rates, and reduced response to conventional therapies [45,46]. This overlap suggests that common molecular drivers, possibly including oxidative stress, may contribute to disease severity across both upper and lower airways.

At the mechanistic level, oxidative stress may enhance type 2 inflammation by promoting eosinophil recruitment and activation through redox-sensitive chemokines. For example, epithelial 15-lipoxygenase-1 promotes the expression of CCL26/eotaxin-3 in nasal polyps [36], facilitating persistent eosinophilic infiltration and reinforcing chronic inflammation and tissue remodeling [42]. Furthermore, ROS-mediated signaling interacts with epithelial-derived cytokines and innate immune pathways, including inflammasome activation, thereby amplifying both allergic and non-allergic inflammatory responses [25,27].

Recent molecular studies have also highlighted the role of oxidative stress in linking metabolic dysregulation and inflammation in CRSwNP. Increased lipid peroxidation in nasal epithelial cells, driven by type 2 cytokines such as IL-4 and IL-13, has been associated with disease severity and epithelial dysfunction, suggesting that oxidative damage extends beyond classical inflammatory pathways to include metabolic reprogramming [26].

Overall, these findings are consistent with a unified model in which oxidative stress may act as a shared mediator bridging allergic inflammation, asthma, and nasal polyp formation. Rather than representing entirely separate disease entities, these conditions may be interconnected manifestations of shared redox-sensitive inflammatory processes, with potential implications for integrated therapeutic strategies targeting both upper and lower airway disease; however, longitudinal human data confirming this model are lacking.

### 3.6. Oxidative Stress Across CRSwNP Endotypes and Populations

CRSwNP is not a single disease entity, and oxidative stress does not appear to follow a uniform pattern across endotypes. In a multicenter study using identical methods, more than 50% of patients with CRSwNP from Europe, Australia, and Japan had a predominantly eosinophilic, type 2-biased endotype, compared with less than 30% of patients from two Chinese centers, where mixed type 1/type 2/type 17 profiles and higher myeloperoxidase levels were common [5]. In East Asia, however, the proportion of eosinophilic disease has been increasing [6]. Direct comparisons of oxidative markers between endotypes are few, small, and derived almost exclusively from East Asian or Turkish centers, which limits their generalizability to Western populations.

In eosinophilic CRSwNP, redox imbalance appears to be dominated by type 2 cytokine-driven lipid oxidation and by phagocyte NADPH oxidase activity. IL-4 and IL-13 induce epithelial ALOX15 and lipid peroxidation programs (SLC27A2/FATP2), whose expression correlates with disease severity in eosinophilic CRSwNP [26,36], and ALOX15-expressing macrophages recruit eosinophils and promote epithelial remodeling [40,47]. Transcriptomic analysis identified NADPH oxidase components (NCF2, NCF4, and RAC2) and HIF1A as oxidative stress-related signature genes upregulated in eosinophilic CRSwNP [28], and metabolomic profiling revealed enrichment of arachidonic and linoleic acid metabolites linked to lipid peroxidation in eosinophilic polyps [48]. Polymorphisms of the catalase and iNOS genes have also been associated with eosinophilic, but not non-eosinophilic, disease [22]. Notably, a loss-of-function ALOX15 variant confers strong protection against nasal polyps [41]; although 15-lipoxygenase-mediated enzymatic lipid oxidation overlaps with, but is not identical to, ROS-mediated oxidative stress, this represents the strongest human genetic evidence to date that a lipid-oxidizing pathway is involved in polyp formation.

In non-eosinophilic CRSwNP, the redox profile appears to be driven to a greater extent by innate and neutrophilic pathways. Heme oxygenase-1 expression was significantly higher in non-eosinophilic than in eosinophilic polyps and was induced in nasal explants by IFN-γ, IL-17A, and IL-27, as well as by type 2 cytokines [21]. NLRP3 inflammasome activation and pyroptosis were present in both endotypes but were more pronounced in non-eosinophilic polyps, with IL-17A rather than IL-5 acting as the main trigger [27]. Metabolomic profiles of non-eosinophilic polyps showed shifts in amino acid and nitrogen metabolism, including increased glutamine and taurine [48], and neutrophil-derived myeloperoxidase, which generates hypochlorous acid, is more abundant in neutrophil-rich CRSwNP [5]. Thus, signal transduction is not unified: ROS converge on shared nodes (NF-κB, NLRP3, and NRF2), but the upstream sources differ, with eosinophil and macrophage NADPH oxidases and 15-lipoxygenase-driven lipid peroxidation predominating in type 2 disease, and neutrophil myeloperoxidase, inflammasome activation, and IL-17 signaling predominating in non-type 2 disease (Figure 1).

From a clinical standpoint, the most severe and refractory phenotypes are eosinophilic CRSwNP, particularly with comorbid asthma or NSAID-exacerbated respiratory disease (N-ERD). In the multicenter JESREC study, blood eosinophilia, ethmoid-dominant disease, asthma, and NSAID intolerance were significantly associated with recurrence after surgery [45], and comorbid CRSwNP contributes to difficult-to-control asthma [46]. Whether oxidative burden is independently associated with these severe phenotypes remains unknown, because no study has compared validated oxidative markers across JESREC or EPOS severity categories or in patients with N-ERD specifically. Likewise, no single oxidative marker can currently be considered the most appropriate. Composite serum indices (TOS, TAS, and OSI) are inexpensive but non-specific and influenced by systemic comorbidities [11,19]; tissue markers such as 4-HNE and 3-nitrotyrosine are more specific to the sinonasal compartment but require tissue sampling [14]; and transcriptomic signatures (e.g., HMOX1, NOS2, SLC27A2, and NADPH oxidase components) are promising for endotyping but remain research tools [26,28]. Future studies should therefore combine a tissue lipid peroxidation marker with a measure of antioxidant capacity and formal endotyping, rather than relying on a single systemic index.

## 4. Discussion

The evidence synthesized in this review is consistent with a model in which oxidative stress represents a shared biological axis linking allergic inflammation, asthma, and chronic rhinosinusitis with nasal polyps (CRSwNP). Rather than acting solely as a downstream consequence, redox imbalance may participate in the initiation, amplification, and persistence of airway inflammation across both upper and lower respiratory tracts.

In allergic diseases, environmental triggers such as allergens and airborne pollutants induce epithelial production of reactive oxygen species (ROS), leading to early oxidative injury and activation of inflammatory pathways [42,49]. Experimental and clinical studies have shown that oxidative stress can accompany and promote allergic sensitization, airway hyperresponsiveness, and chronic inflammation, suggesting, mainly on the basis of experimental data, a contributory role in disease development [42,43]. This concept is further supported by evidence showing that ROS directly activate transcription factors such as NF-κB and enhance type 2 immune responses, thereby amplifying cytokine production and inflammatory cell recruitment [9,42].

In asthma, oxidative stress is now recognized as a key contributor to airway remodeling and disease persistence. Increased ROS production, derived from both inflammatory cells and environmental exposures, has been associated with epithelial damage, mucus hypersecretion, and structural changes in the bronchial wall [43,44,50]. Importantly, oxidative stress not only reflects inflammation but may also reinforce it through a feed-forward mechanism in which ROS generation and immune activation perpetuate each other.

A similar redox imbalance has been demonstrated in the upper airways, particularly in patients with CRSwNP. Studies have shown increased oxidative stress markers in nasal polyp tissue and epithelial cells, accompanied by mitochondrial dysfunction and altered cellular metabolism [14,23,24]. These alterations closely resemble those observed in asthma, reinforcing the concept that upper and lower airway diseases share common molecular drivers.

Environmental exposure further strengthens this link. Air pollutants such as particulate matter (PM2.5), ozone, and nitrogen dioxide are potent inducers of oxidative stress and have been associated with both asthma exacerbations and chronic rhinosinusitis. These agents promote epithelial barrier disruption and inflammatory activation through ROS-mediated mechanisms, contributing to disease severity and progression [49]. Similarly, seasonal air pollution has been associated with reduced antioxidant status and increased airway reactivity in patients with rhinitis, with distinct patterns in allergic and non-allergic rhinitis [51], suggesting that oxidative stress is an early and persistent feature across the airway disease spectrum.

At the tissue level, oxidative stress may contribute to the structural changes characteristic of nasal polyps. Increased levels of oxidative products and reduced antioxidant defenses have been repeatedly reported in sinonasal tissues, supporting a role for redox imbalance in polyp formation and growth [12,14,19]. These findings align with the observation that patients with asthma and CRSwNP often exhibit more severe disease phenotypes [45,46], suggesting that shared oxidative mechanisms may underlie this clinical overlap.

From a mechanistic perspective, oxidative stress acts at multiple levels of the inflammatory cascade. It enhances epithelial permeability, promotes cytokine release, activates innate immune pathways, and modulates adaptive immune responses. In parallel, impaired antioxidant systems fail to counterbalance these effects, leading to a persistent pro-oxidative microenvironment that sustains chronic inflammation.

Taken together, these data support viewing oxidative stress not merely as a secondary by-product of inflammation but as a plausible integrative factor linking allergy, asthma, and CRSwNP. If confirmed, this unified perspective would have important clinical implications, as it suggests that targeting redox imbalance may provide therapeutic benefits across the spectrum of airway disease.

A critical appraisal of the evidence, however, reveals important limitations. First, virtually all human studies are cross-sectional case–control comparisons with small samples, typically 20–40 patients per group, which frequently use patients undergoing septal or turbinate surgery rather than healthy individuals as controls and rarely adjust for smoking, atopy, asthma, previous surgery, or corticosteroid exposure, all of which may influence redox markers (Table 1). Second, measurements were usually performed in a single compartment and at a single time point, so temporality cannot be established. Third, several findings, such as reduced antioxidant enzyme activity, have not been replicated consistently, and composite indices such as TOS and OSI lack specificity.

Regarding causality, three lines of evidence suggest that oxidative stress is more than an epiphenomenon, although none is conclusive. First, experimental manipulation: genetic deletion of NRF2 aggravates, and pharmacological NRF2 activation or mitochondrial ROS scavenging attenuates, sinonasal inflammation and barrier dysfunction in animal and cell models [23,31,32,33]. Second, human genetics: a loss-of-function ALOX15 variant is strongly protective against nasal polyps [41], and antioxidant enzyme polymorphisms have been associated with eosinophilic disease [22]; germline variants precede disease onset and are therefore less susceptible to reverse causation, although 15-lipoxygenase also acts through enzymatic lipid mediators. Third, biological gradient: some redox markers scale with disease severity or predict recurrence [13,18,26]. Conversely, the partial normalization of oxidative markers after anti-inflammatory treatment, such as corticosteroids or dupilumab [13,26], is equally compatible with oxidative stress being downstream of inflammation. The most plausible interpretation is therefore that oxidative stress acts as an amplifier within a self-sustaining inflammatory loop rather than as an independent initiating cause, a hypothesis that requires longitudinal and interventional studies to be tested.

Furthermore, although preliminary data suggest endotype-specific oxidative signatures, direct comparisons between eosinophilic and non-eosinophilic disease, and between Western and East Asian populations, remain scarce.

Where might antioxidant therapy fit in the future? On current evidence, antioxidants cannot be recommended for the treatment or prophylaxis of CRSwNP. Should efficacy be demonstrated, the most rational initial setting would be as an adjunct in patients with a documented high oxidative burden, for instance, those with recurrent or uncontrolled type 2 disease after surgery or with heavy oxidant exposure such as smoking, rather than as primary prophylaxis in unselected individuals, for whom there is no supporting evidence. Endotype-guided approaches are also conceivable, such as targeting NADPH oxidase, ALOX15, or FATP2-driven lipid peroxidation in eosinophilic disease, and NLRP3-dependent pyroptosis in non-eosinophilic disease [25,26,27,40]. Future trials should stratify patients by endotype and include validated redox biomarkers as secondary endpoints.

Future research should focus on longitudinal studies integrating molecular, clinical, and environmental data, ideally stratified by endotype and ethnicity, to better define the role of oxidative stress in disease progression. In addition, the development of standardized oxidative biomarkers and adequately powered randomized trials of targeted antioxidant therapies may represent a crucial step toward the personalized management of airway inflammatory diseases.

## 5. Conclusions

This narrative review highlights the growing evidence implicating oxidative stress in the pathophysiology of chronic rhinosinusitis with nasal polyps (CRSwNP). Oxidative stress, driven by an imbalance between reactive oxygen species (ROS) production and antioxidant defense mechanisms, appears to contribute to epithelial barrier dysfunction, amplification of type 2 inflammatory responses, and tissue remodeling characteristic of nasal polyps. Key molecular pathways, including NF-κB activation, NLRP3 inflammasome signaling, and insufficient NRF2-dependent antioxidant responses, emerge as plausible links between redox imbalance and chronic inflammation, and oxidative signatures appear to differ between eosinophilic and non-eosinophilic endotypes. Furthermore, oxidative stress may represent a shared mechanism bridging allergic rhinitis, asthma, and CRSwNP within the united airways disease framework. However, current human evidence is mainly cross-sectional and does not establish causality, and antioxidant strategies are supported almost exclusively by preclinical data. Standardized oxidative biomarkers, longitudinal studies, and well-designed, endotype-stratified clinical trials are needed before redox biology can be integrated into the personalized management of CRSwNP.

## Data Availability

No new data were created or analyzed in this study. Data sharing is not applicable to this article.
